# Essential Palatal Tremor Following COVID-19 Vaccine: A Case Report

**DOI:** 10.7759/cureus.70420

**Published:** 2024-09-29

**Authors:** Khalil Toumi, Yahya Naji, Sara Laadami, Nawal Adali

**Affiliations:** 1 Department of Neurology, CHU (Centre Hospitalier Universitaire) Souss Massa, Agadir, MAR; 2 Department of Neurology, Agadir University Hospital, Agadir, MAR

**Keywords:** clonazepam, covid-19 vaccine, guillain-mollaret triangle, inferior olive, palatal tremor

## Abstract

Palatal tremor (PT) is an infrequent disorder characterized by abnormal movement distinguished into symptomatic palatal tremor (SPT) and essential palatal tremor (EPT). SPT can have various causes, including damage to the Guillain-Mollaret triangle and hypertrophy of the inferior olive. In contrast, EPT is often associated with clicking sounds in the ears with normal imaging results and may have a functional origin. We describe a case of a 20-year-old girl presenting with clinical symptoms of PT two days after receiving the SARS-CoV-2 AstraZeneca vaccine. The patient had persistent audible clicks associated with rhythmic contractions of the soft palate. Complete resolution of symptoms occurred two days after the administration of oral clonazepam. This is the first known case of PT caused by the SARS-CoV-2 AstraZeneca vaccine.

## Introduction

Palatal tremor (PT), formerly known as palatal myoclonus, is a rare movement disorder characterized by rhythmic, usually involuntary, jerky movements of the soft palate [1]. PT is typically divided into two clinical and etiological subtypes: essential palatal tremor (EPT) and symptomatic palatal tremor (SPT) [1,2].

EPT remains poorly understood, with various theories suggesting it may be caused by a central generator, peripheral factors, or even psychogenic or voluntary mechanisms [3]. In contrast, SPT is caused by lesions in a specific area of the brainstem known as the Guillain-Mollaret triangle (GMT). It is characterized by hypertrophic olivary degeneration, visible on MRI. This tremor can also be triggered by external factors, such as certain medications or vaccines [4,5].

Indeed, although SARS-CoV-2 vaccines are largely effective in combating the pandemic, they have been associated with neurological side effects in some cases. While these effects are often acute and transient, it is important to note that they can also be severe and, in rare cases, fatal [6]. In this case, we report a patient who developed PT following the administration of the first dose of the SARS-CoV-2 AstraZeneca vaccine.

## Case presentation

Patient information

We present the case of a 20-year-old female patient with no significant pathological history who presented with an audible click in her ears two days after receiving the first dose of the AstraZeneca SARS-CoV-2 vaccine (May 2021) without other associated symptoms, including no persistent headaches, visual fog, dysphagia, dysarthria, fever, weight loss, or other neurological or auditory symptoms. She also had increasing difficulty concentrating on daily tasks.

Clinical findings

The otological examination showed that the tympanic membranes were normal, and that hearing was also intact, as confirmed by audiological tests, including tonal audiometry and impedance. During the examination of the palate, rhythmic, involuntary, and symmetrical contractions of the soft palate were observed, oscillating at a frequency of 40 to 50 clicks per minute (Video 1). These contractions produced an audible click in the ears and persisted during sleep as well as during phonation. It is important to note that the lips, tongue, larynx, and diaphragm were not affected by these contractions. Furthermore, neurological and systemic examinations revealed no notable abnormalities.

**Video 1:** Patient with rhythmic, involuntary contractions of the soft palate after receiving the first dose of the AstraZeneca SARS-CoV-2 vaccine.

Diagnostic assessment

**Figure 1:**
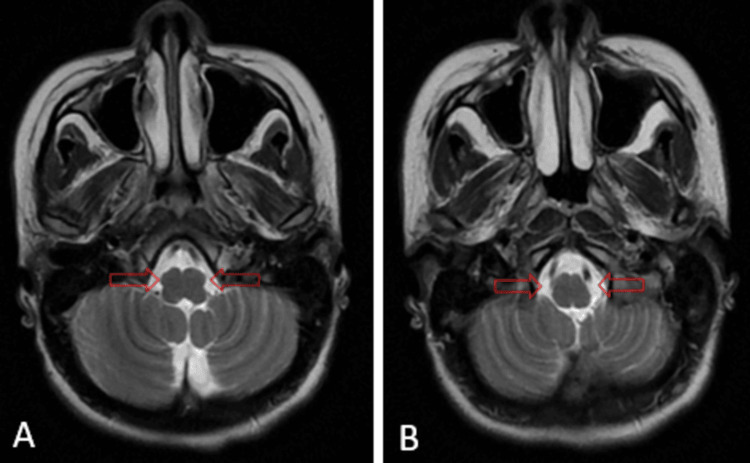
(A, B) Magnetic resonance imaging. Axial T2-weighted imaging did not show an increased signal of the inferior olivary nucleus (red arrows) or enhancement following gadolinium injection.

All of the blood test results, including liver, renal, and thyroid function, were normal. There were no significant lesions observed during brain magnetic resonance imaging (MRI), notably no inferior olivary hypertrophy (Figure 1).

Diagnosis

We considered the diagnosis of vaccine-induced EPT due to the short duration between vaccine administration and the onset of tremors, the absence of other associated neurological or auditory signs, and the normality of biological assessments and brain MRI.

Therapeutic intervention and follow-up

She was effectively treated with oral clonazepam at a dose of 1 mg per day two days after her admission. The patient declined treatment with botulinum toxin injections. Over the six-month follow-up period, a partial recurrence of the tremor was observed when her medication was reduced, requiring a return to the initial clonazepam dosage, along with the introduction of a new centrally acting drug, sodium valproate (400 mg/day). No neurological complications were observed during follow-up.

## Discussion

PT is an uncommon condition that was first described by Politzer in 1878 [2]. Initially called "palatal myoclonus," it was renamed "palatal tremor" at the First International Congress of Movement Disorders in 1990 [3]. PT is characterized by rhythmic movements of the soft palate, rapid and rhythmic involuntary movements of the soft palate, and the muscles around the auditory tube. This condition causes a clicking sound in the ear, which can be disturbing for the sufferer [3]. Two distinct forms of this condition have been identified, namely, an essential form and a symptomatic form [7].

SPT is linked to an identifiable cause, typically a lesion in the GMT. This triangle includes the dentate nucleus, red nucleus, and inferior olivary nucleus, forming a neural pathway that can lead to hypertrophic olivary degeneration, which becomes visible on MRI around four to six months after the initial injury [4]. This hypertrophic olivary degeneration is considered a key factor in the pathophysiology of SPT, leading to synchronized oscillations of neurons in the inferior olivary nucleus [5]. Common causes of SPT include infarction (hemorrhagic or ischemic), tumors, vascular malformations, medications, and demyelination [4].

In contrast, there is no demonstrable cause or associated physical or radiological signs in EPT, and its natural course remains unknown, mainly because it is very rare, and its nature is most likely heterogeneous [3].

The pathophysiology of PT can be explained by several distinct origins. The central origin is characterized by functional MRIs revealing potential tremor generators in the inferior olive and brainstem, although the final pathways for these two types of tremors differ. Regarding the peripheral origin, patients with EPT often show inflammation of the oral and nasal mucosa, and upper respiratory tract infections can trigger this condition. As for the voluntary origin, some individuals may voluntarily contract the tensor veli palatini (TVP) muscle, the main muscle involved in EPT. Finally, the psychogenic origin is related to common triggers such as emotional stress or past trauma [4].

Symptomatically, SPT presents with cerebellar dysfunction, ataxia, oscillopsia, and neurological signs such as nystagmus, dysarthria, and ataxia, whereas EPT is mainly characterized by the presence of auricular clicks without other neurological abnormalities, which generally disappear during sleep but persist in the case of SPT [8].

AstraZeneca’s SARS-CoV-2 vaccine, also called Vaxzevria, is designed to protect against the virus responsible for COVID-19. Utilizing viral vector technology, the vaccine has been linked to several neurological side effects, including Guillain-Barré syndrome (GBS), cerebral venous sinus thrombosis (CVST), demyelinating disorders of the central nervous system, Bell's palsy, phantosmia, encephalopathy/encephalitis, Tolosa-Hunt syndrome, seizure disorders, and acute aseptic meningoencephalitis associated with Sweet's syndrome [9].

In the absence of any other clear causes, we considered vaccine-induced EPT in our patient, even though the clicking sound continued during sleep. As far as we know, this is the first reported case of PT linked to the SARS-CoV-2 vaccine.

A range of pharmacological agents are used in the treatment of PT, including anticonvulsants, benzodiazepines, anticholinergic agents, calcium channel blockers such as flunarizine, serotonin agonists (like sumatriptan), nootropics (such as piracetam), placebos, and botulinum toxin [10].

The treatment of EPT may also include cognitive-behavioral therapy. For SPT, treatment depends on the underlying cause, ranging from medical management to surgery for the removal of the responsible lesion [4].

## Conclusions

PT is an uncommon movement disorder distinguished by rhythmic contractions of the soft palate. It can be divided into two types: idiopathic and symptomatic. This case is the first to document PT arising after receiving the AstraZeneca SARS-CoV-2 vaccine. This finding highlights the necessity of considering PT in the differential diagnosis for patients who develop auditory symptoms following vaccination.
